# Diameter Control of GaSb Nanowires Revealed by *In Situ* Environmental Transmission Electron Microscopy

**DOI:** 10.1021/acs.jpclett.3c01928

**Published:** 2023-08-11

**Authors:** Mikelis Marnauza, Robin Sjökvist, Sebastian Lehmann, Kimberly A. Dick

**Affiliations:** † Centre for Analysis and Synthesis, Lund University, 22100 Lund, Sweden; ‡ NanoLund, Lund University, 22100 Lund, Sweden; § Solid State Physics, Lund University, 22100 Lund, Sweden

## Abstract

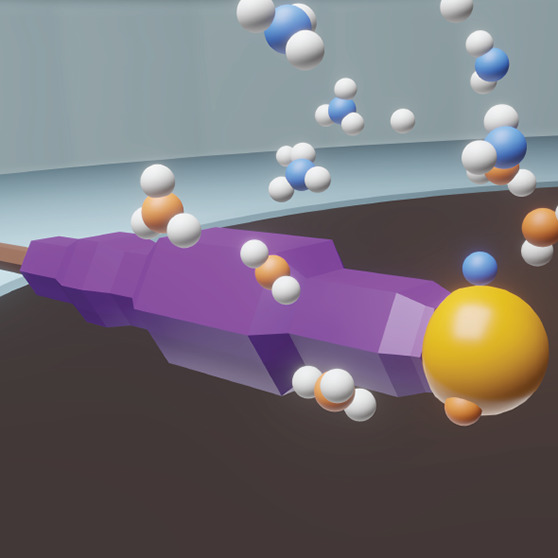

Several nanowire properties are strongly dependent on their diameter, which is notoriously difficult to control for III–Sb nanowires compared with other III–V nanowires. Herein environmental transmission electron microscopy is utilized to study the growth of Au nanoparticle seeded GaSb nanowires *in situ*. In this study, the real time changes to morphology and nanoparticle composition as a result of precursor V/III ratio are investigated. For a wide range of the growth parameters, it is observed that decreasing the V/III ratio increases the nanoparticle volume through Ga accumulation in the nanoparticle. The increase in nanoparticle volume in turn forces the nanowire diameter to expand. The effect of the V/III ratio on diameter allows the engineering of diameter modulated nanowires, where the modulation persisted after the growth. Lastly, this study demonstrates the observed trends can be reproduced in a conventional *ex situ* system, highlighting the transferability and importance of the results obtained *in situ*.

III–Sb nanowires (GaSb, InSb, AlSb, and their ternary combinations) are important materials for potential uses in applications such as quantum electronics, thermoelectrics, and sensing.^1,2^ This is due to their excellent electrical properties including high carrier mobility and narrow band gap. Regarding carrier mobility the most notable examples are InSb and GaSb, which display the highest electron and hole mobility among the III–V materials, respectively.^3^

Despite the superior properties of this material platform, little research has been conducted in comparison to other III–V materials such as III–As nanowires. This is due to the low vapor pressure and surfactant properties of elemental Sb which negatively impact the ability to attain controlled growth of III–Sb nanowires.^4^ In addition, Sb is known to have a higher solubility in Au compared to other group V elements.^1^ These material-specific properties often lead to challenges in controlling fundamental aspects of the growth, such as direct nucleation on crystalline substrates. However, the growth of III–Sb nanowires can be facilitated by using a III–As nanowire stem, forming a heterostructure. The switch to III–Sb is typically accompanied by a dramatic increase in diameter relative to the original III–V stem dimensions, which complicates the growth of diameter-specific III–Sb nanowires.^1,2,5^

Nanowire diameter has been shown to have a significant impact on the electrical, thermal, and optical properties of nanowires.^6−11^ Several studies have also demonstrated that modulated nanowire diameters can enhance properties such as absorption by more efficient light trapping and enhanced thermoelectric conversion. This highlights the importance of fundamental knowledge connecting the growth conditions and resulting nanowire morphology.^12−14^ The correlation between growth conditions and nanowire diameter in GaSb and InSb nanowires has been explored in earlier studies.^1,2,5^ However, due to the inability to observe growth dynamics and determine seed particle composition during growth, a crucial step connecting the precursor pressures used and the resulting nanowire diameter is missing. This makes the observed trends difficult to interpret and does not provide a general understanding of the interplay between vapor, liquid, and solid phases.

The introduction of environmental transmission electron microscopes (ETEMs) has allowed the exploration of some of the previously hidden aspects of nanowire growth dynamics in real time.^15−21^ Importantly, nanowires have in some circumstances been shown to exhibit unconventional growth modes that differ from the traditional view.^22,23^*In situ* growth has also proven to be an invaluable technique in developing new methods and strategies to circumvent known problems in conventional *ex situ* nanowire growth systems, which has most notably been demonstrated in Si nanowire growth.^16,17^ The strength lies in that the surrounding growth conditions can be changed in real time based on the observations made, and the effects on the growth are immediate, which means that conclusions about the growth dynamics can quickly be drawn. By monitoring the growth and how it changes with the growth conditions, it is also easier to make rational decisions about how to adjust the growth conditions to achieve a specific result. Thus far, *in situ* studies of III–V nanowires have mostly focused on III–As and III–P nanowires (along with their ternary combinations), investigating aspects such as nanowire nucleation, droplet and nanowire composition during growth, crystal phase stability, and the layer-by-layer growth process.^24−29^

In this work, we demonstrate the first study of III–Sb nanowires in an *in situ* ETEM, using Au-seeded GaAs–GaSb nanowire heterostructures as our model system. *In situ* measurement of particle composition reveals that stable growth of GaSb nanowires can be achieved with Ga concentrations as high as 94 at. % and as low as 66 at. % in the droplet. This composition can be tuned by varying the individual precursor partial pressures and determines the droplet volume, which, in turn, influences the resulting nanowire diameter. We demonstrate that adjusting the precursor partial pressures allows the nanowire diameter to be tuned within a range of 45–100 nm for a nominal Au seed particle diameter of 30 nm. Furthermore, we show that the observations from *in situ* nanowire growth can be transferred to a conventional system by demonstrating similar nanowire diameter tunability as a function of V/III ratio for Au-seeded GaAs–GaSb nanowire heterostructures grown *ex situ*.

The GaSb segments were grown as a part of GaAs–GaSb nanowire heterostructures using nominally 30 nm diameter Au particles as the seeds, as shown in Figure 1a. X-ray energy dispersive spectroscopy (XEDS) showed that the switch between GaAs and GaSb involved the formation of ternary GaAs_*x*_Sb_1–*x*_ transition regions which, in general, were determined to be <50 nm in length (not highlighted in Figure 1a). The full details describing the nucleation and growth can be found in the Experimental Methods section.

**Figure 1:**
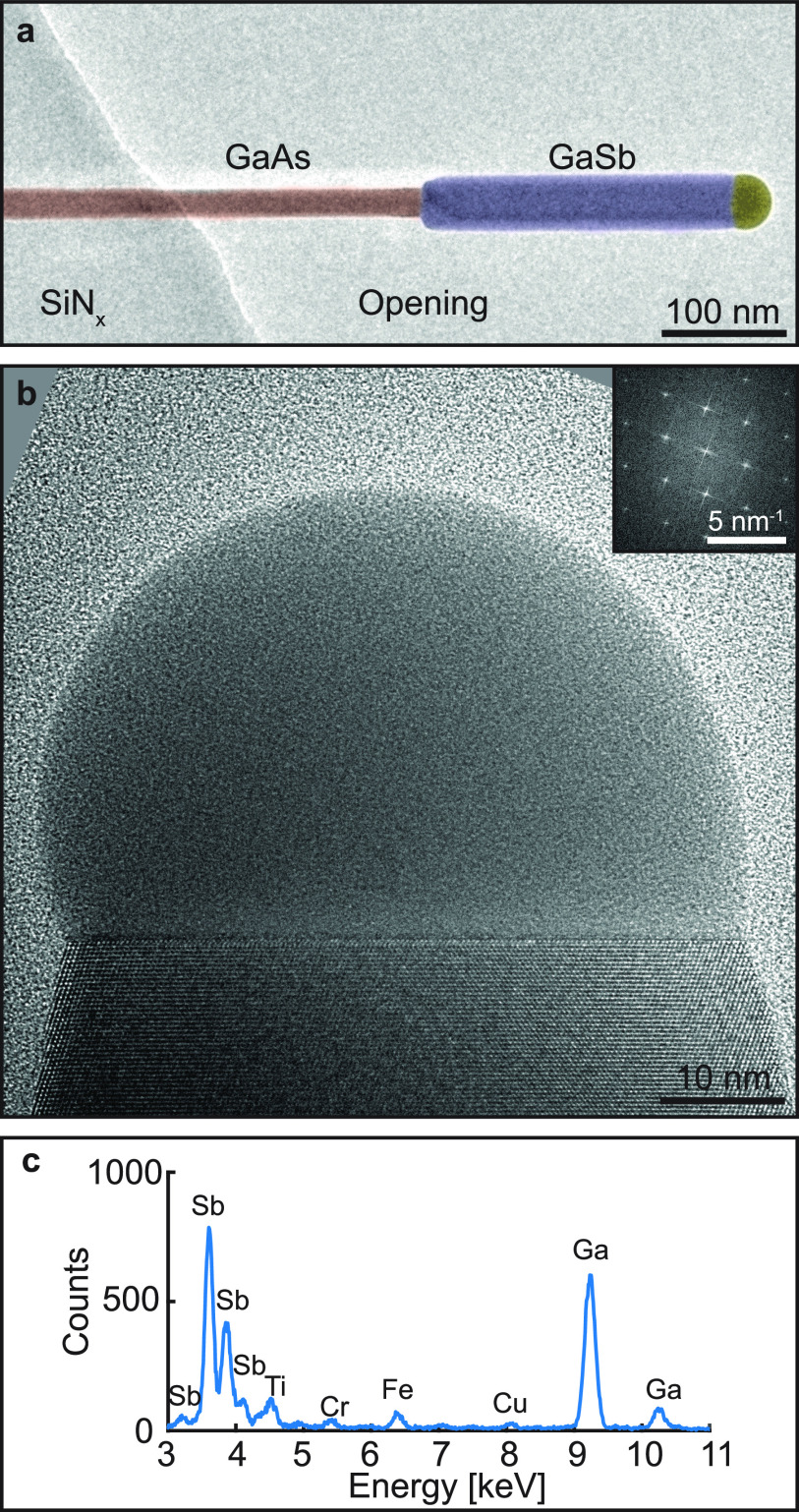
***In situ* growth of Au-seeded GaSb nanowires**. (a) False-colored image of a typical nanowire as viewed in ETEM. The GaAs nanowire was grown from the SiN_*x*_ film into one of the openings of the MEMS chip, where the gaseous environment was changed in order to initiate GaSb growth. (b) A GaSb nanowire grown over the SiN_*x*_ film, showing the atomically resolved ZB crystal structure. The inset shows an FFT of the solid crystal, indicating that the crystal structure is pure ZB. (c) Part of the XEDS spectrum related to the nanowire shown in (b), showing elements present in the nanowire as well as some peaks originating from different parts of the setup.

In Figure 1b, a high-resolution transmission electron microscope (HRTEM) micrograph depicting a typical Au-seeded GaSb nanowire aligned to the ⟨11̅0⟩ zone axis is shown, as determined from the fast Fourier transform (FFT) of the solid phase shown in the inset. The crystal structure of the nanowires was determined to be zincblende (ZB) as evident from the HRTEM image and FFT, as expected from previous studies involving GaSb nanowires.^30−33^ The XEDS spectrum corresponding to the nanowire in Figure 1b is shown in Figure 1c. Quantification of the XEDS data indicated a composition of 47 at. % Ga and 53 at. % Sb confirming that the nanowire was GaSb, with the other minor detected peaks originating from the holder, gas injector, and MEMS chip used as the substrate. The minor deviation from a perfect stoichiometric ratio is attributed to the electron channelling effect, which is especially pronounced when XEDS spectra are acquired under near zone axis conditions.^34^

To gain insight into the formation of the Au-seeded GaSb nanowires, two separate experimental series were performed by varying the flows of the two precursors independently after the initial formation of the transition region. During the investigation, XEDS was used to monitor the composition of the seed particle, while micrographs were used to monitor the diameter and general behavior of the nanowire growth.

One of the most important pieces of information required to control the nanowire growth is understanding how growth conditions affect the liquid particle composition, as it dictates the nanowire growth process.^26^ Therefore, in this section, the nanoparticle composition during the growth of GaSb will be discussed. Figure 2a shows the measured concentrations of Au, Ga, and Sb in at. % as a function of the gas phase V/III ratio. The V/III ratio was changed by altering the influx of trimethylantimony (TMSb) or trimethylgallium (TMGa) supplied to the microscope while keeping the other precursor influx constant. In the following, these will be referred to as TMSb and TMGa series and are represented by filled and striped shapes, respectively, in Figure 2a. It is worth noting that the XEDS data shown in Figure 2 are presented only for conditions where growth was observed. Outside of the presented range, the growth was either too slow (no change within 20 min of observation) or unstable (leading to particle displacement from the top facet). The partial pressures of precursors corresponding to the used growth conditions can be found in Supporting Information SI-1, while the XEDS quantification results can be viewed in Supporting Information SI-2.

**Figure 2:**
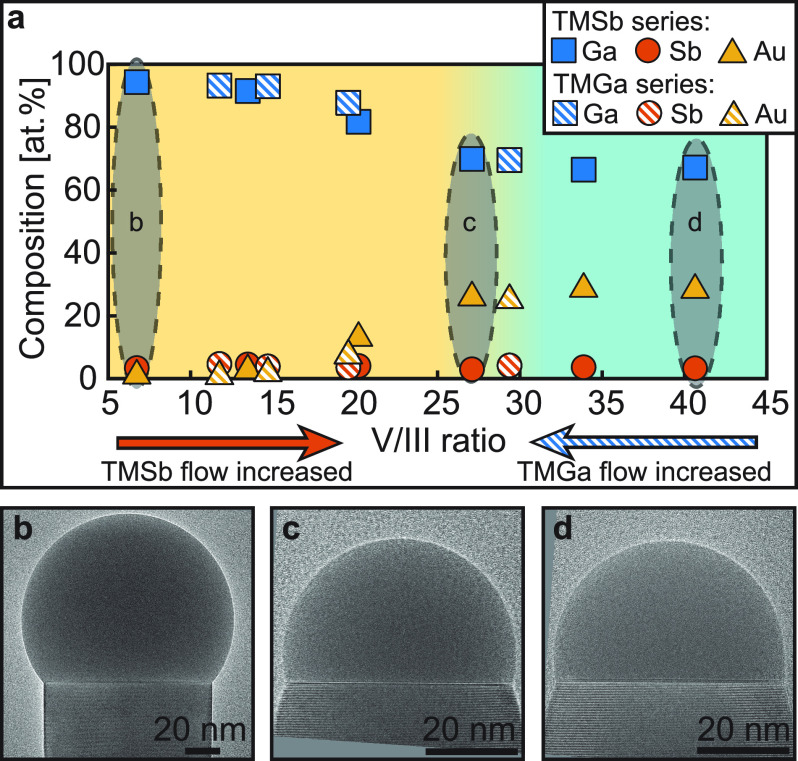
**Droplet composition and morphology as a function of the V/III ratio**. (a) Nanoparticle composition as a function of V/III ratio for the TMSb and TMGa flow series depicted as filled and striped shapes, respectively. The yellow and cyan shaded regions represent the dynamic and constant droplet composition regimes, respectively. (b–d) Images of nanowires at low (b), medium (c), and high (d) V/III ratio, as indicated by the shaded areas in (a), labeled “b”, “c”, and “d”, respectively.

We start by examining the TMSb series (filled shapes). As can be seen from Figure 2a, at the lowest V/III ratio (lowest TMSb flow) we measured the Ga concentration in the particle to be approximately 94 at. %, while the Sb concentration was 4 at. % with the remaining 2 at. % corresponding to Au (see Supporting Information SI-2). Although the accuracy of the XEDS quantification in our system (at the used growth temperature) is assumed to be in the order of 1–2 at. %,^26^ we were always able to observe characteristic X-ray peaks belonging to Sb as shown in Supporting Information SI-3. Increasing the V/III ratio resulted in a steady decrease of the Ga concentration in the particle from the originally observed 94 to 66 at. %, with the Sb concentration always measured between 3 and 4 at. %. As a consequence of the decrease in Ga concentration we also observed a change in droplet volume as evident from Figure 2b–d. Interestingly, the droplet composition did not change monotonically with an increase in TMSb flow, allowing us to distinguish two regimes. These are shown in Figure 2a as the yellow and cyan shaded areas, which will be further referred to in the text as the “dynamic composition” (yellow) and “stable composition” (cyan) regimes. In the dynamic composition regime, we observe a decrease of Ga concentration in the particle with increasing V/III ratio, which causes a decrease of droplet volume. The change in droplet volume leads to a dramatic reduction in the nanowire diameter as is evident from Figure 2b,c. In the stable composition regime, however, the Ga concentration in the particle remains constant with increasing V/III ratio (within the estimated error of XEDS), which results in a stable droplet volume and nanowire diameter, as seen in Figure 2c,d.

A trend similar to that of the TMSb series was also observed when we examined the TMGa series (TMGa flow was changed for constant TMSb flow) as seen in Figure 2a, shown with the striped shapes. Here we observe a Ga concentration in the particle above 90 at. % which gradually dropped to about 69 at. % when the V/III ratio was increased, while the Sb concentration remained unaffected at about 3–4 at. %. As for the TMSb series, the reduction in Ga concentration was accompanied by a reduction of droplet volume. Based on our measurements we have determined that stable axial growth of Au-seeded GaSb nanowires occurs when the Ga concentration in the nanoparticle is in the range of 66–94 at. %.

When the nanoparticle composition study presented here is compared to similar studies conducted on other Au-seeded III–Vs such as GaAs nanowires, stark differences emerge. The measured Ga concentration in the particle during growth of Au-seeded GaSb nanowires is significantly higher than that in Au-seeded GaAs nanowire growth, where the Ga concentration has been measured to be in the range of 25–55 at. %^26^ at the same growth temperature. Furthermore, the group V element concentration differs significantly. For Au-seeded GaAs growth the As concentration is too low to be measured by XEDS; based on thermodynamic calculations, it is estimated to be between 0.01 and 1 at. %.^26^ The differences in group V element concentration in the particle are expected to be a direct consequence of elemental As and Sb properties. Arsenic is known to have a high vapor pressure and low solubility in the liquid Au–Ga alloy, whereas Sb has a relatively low vapor pressure and a comparatively high solubility.^1,26,35−37^ The differences in Ga concentration are more difficult to explain, and several theories have been proposed. It has been reported in *ex situ* studies that during GaSb nanowire growth the Ga concentration is generally higher than that during GaAs nanowire growth.^1^ In some of the studies the authors have attributed this phenomenon to a lack of pseudobinary tie-lines to less Ga-rich phases in the Au–Ga–Sb ternary phase diagrams meaning that solidification of GaSb from the liquid Au–Ga–Sb alloy is possible only at high Ga concentrations (≥50 at. %).^1,30^ In other works it has been speculated that the addition of Sb to the system increases the equilibrium concentration of the group III species in the Au particle, compared to arsenide growth, in turn decreasing the supersaturation.^38,39^ This suggests that significantly higher Ga concentrations are required to achieve a high enough supersaturation to facilitate new layer nucleation during GaSb growth compared to GaAs growth. This fits well with the measured droplet composition during growth of GaSb in this study and previous works on GaAs growth *in situ*.^26^

In addition to the droplet volume expansion for decreasing the V/III ratio discussed earlier, we also observed an increase in nanowire diameter, which the nanowire adopted in order to accommodate the larger volume droplet on the liquid–solid (LS) interface. The nanowire diameter measured at the LS interface as a function of V/III ratio for both flow series is depicted in Figure 3a. Here we have plotted diameter only for growth conditions where steady state axial growth was observed; i.e., the nanowire did not kink or stop axial growth.

**Figure 3:**
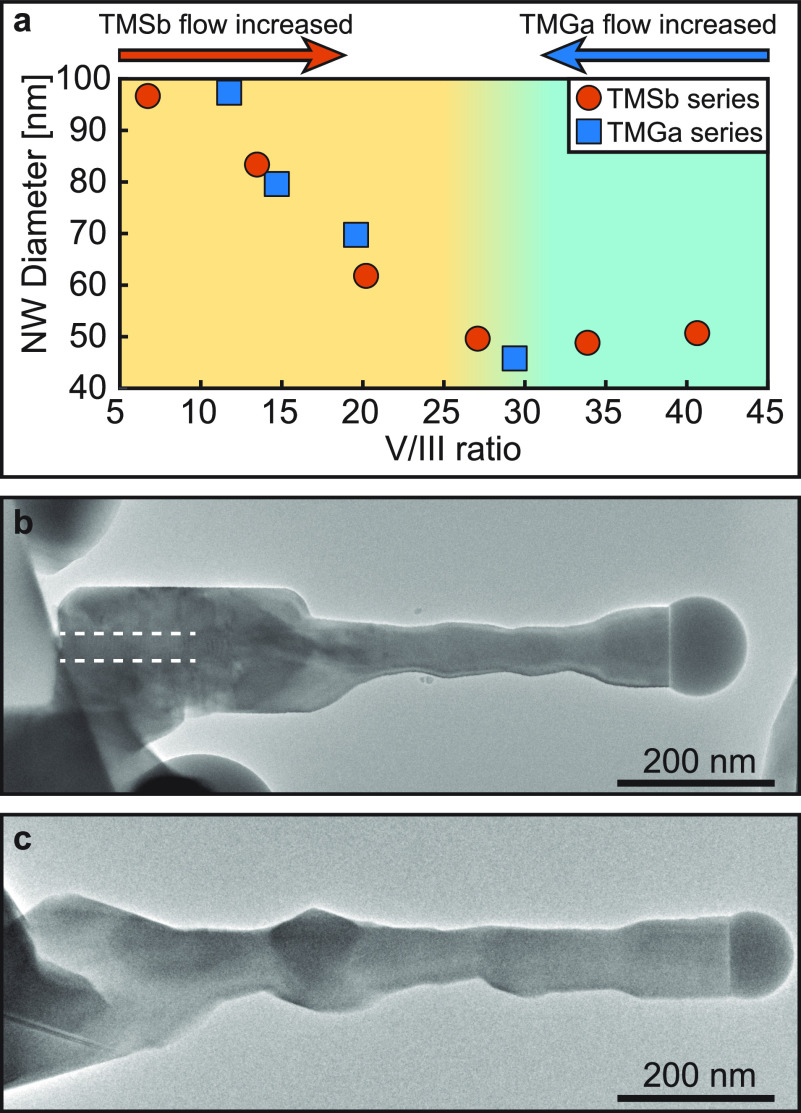
**Nanowire morphology as a function of the V/III ratio**. (a) Nanowire diameter as a function of V/III ratio for the TMSb and TMGa flow series. (b) The resulting nanowire after recording the TMSb series. (c) The resulting nanowire after recording the TMGa series. Note that both nanowires in (b) and (c) retain the diameter after the growth at different V/III ratios resulting in diameter modulated structures.

As can be seen from Figure 3a, astonishingly, nanowire diameter can be tuned across a span of approximately 45–100 nm by adjusting either of the precursor flows. The overview TEM images of GaSb nanowires after the TMSb and TMGa flow series are displayed in Figure 3b and c, respectively. From the images it can be observed that the axial growth dominates the growth process thus resulting in diameter modulated nanowires. The nanowires retained the diameter modulation throughout the experiment, which was on the order of a few hours. The approximate positions along the length of the nanowires where different growth conditions were used are shown in Supporting Information SI-4. Although the nanowire diameter response to either precursor flow change was similar in GaSb nanowires, we observed significant overgrowth of the GaAs segment for the TMSb series at the stable composition regime (cyan region in Figure 3a). This is highlighted by the white dashed outline in Figure 3b displaying the original thickness of the GaAs segment (a higher magnification image showing the transition region can be viewed in Supporting Information SI-5).

To determine whether the trends observed *in situ* are transferable to more standard *ex situ* growth systems, we performed growth of Au-seeded GaAs–GaSb nanowire heterostructures in a conventional metal–organic chemical vapor deposition (MOCVD) system. Further details of the experimental setup can be found in the Experimental Methods section. The growth conditions were chosen such that the GaSb segment diameter under the reference conditions (V/III ratio = 2.0) would closely match the diameter recorded in the *in situ* growth experiments (V/III ratio = 27.1). The partial pressures of both TMGa and TMSb used in *ex situ* growths can be found in Supporting Information SI-6. The results of these experiments are summarized in Figure 4 with scanning electron microscope (SEM) overview images of the as-grown nanowires demonstrated in panels a–d grown at different V/III ratios. Similar to the *in situ* growth performed as a part of this study and previous *ex situ* studies, the nanowires undergo a significant diameter increase when the GaSb segment growth is started as can be seen from the SEM images (a–d).^1,2,5^ The GaSb segment diameter dependence on the V/III ratio is depicted in Figure 4e and shows a trend very similar to that observed during the *in situ* growths. At high V/III ratio the nanowire diameter does not significantly change, whereas below a critical V/III ratio (herein somewhere between a V/III ratio of 1.0 to 2.0) a gradual diameter increase can be observed. Growth at a V/III ratio lower than 0.5 by further reducing the TMSb flow was not possible due to technical limitations; however, the observed diameter increase with lowered V/III ratio is expected to continue similarly to what was observed *in situ*. During the *ex situ* growth, the TMSb flow was changed midway through the growth of the GaSb segment in an attempt to recreate the diameter modulated structures observed *in situ* (Figure 3b,c). In contrast to the *in situ* growth, this resulted in a homogeneous nanowire diameter. This could be due to several different factors such as the difference in total operating pressures, availability of chemical species in the *in situ* and *ex situ* reactors, as well as growth temperatures. Therefore, we speculate that, in order to achieve diameter modulated GaSb nanowires *ex situ*, careful tuning of growth conditions including the growth temperature is required.

**Figure 4:**
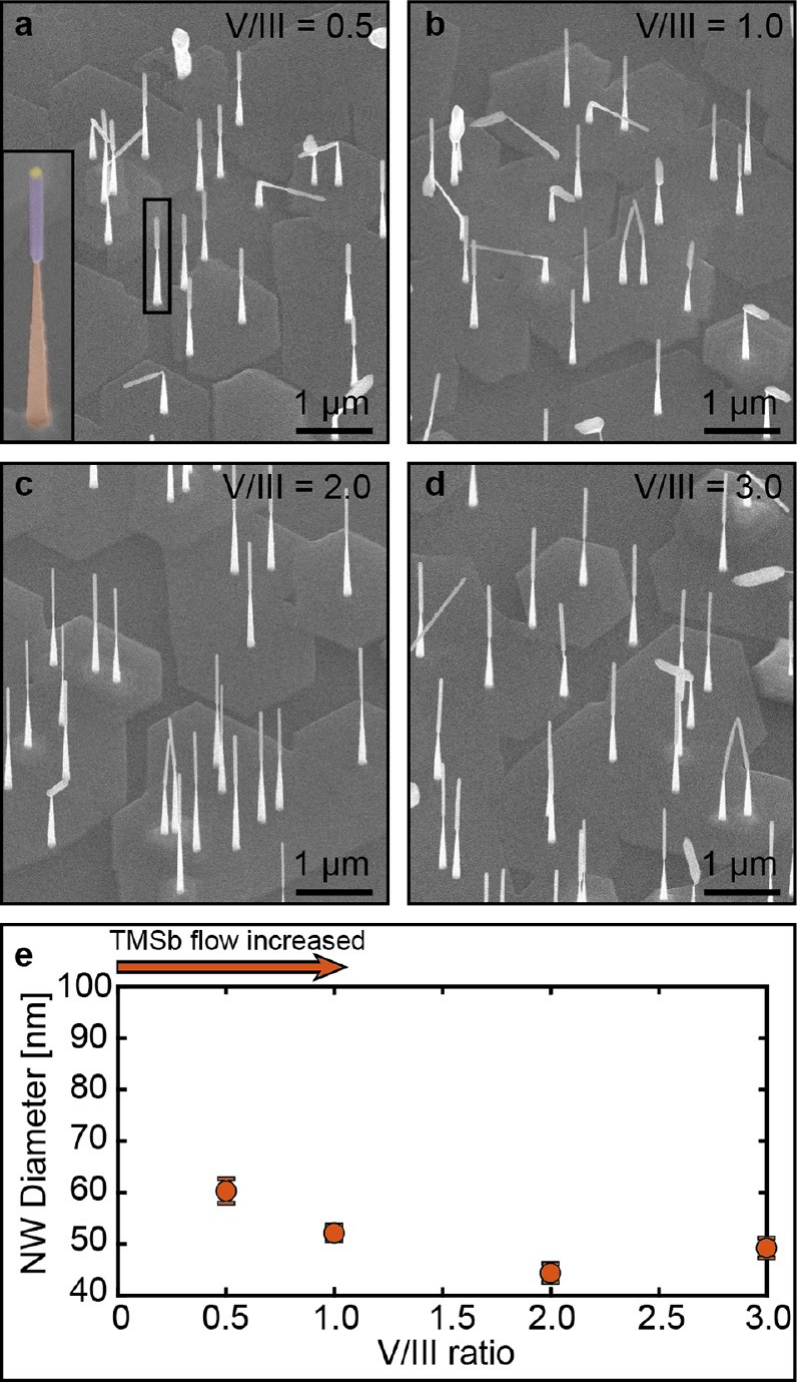
**Nanowire dimensions as a function of the V/III ratio grown in a conventional MOCVD setup**. (a–d) 30° tilted view SEM images of Au-seeded GaAs–GaSb nanowires grown at different V/III ratios. The inset in (a) shows a magnified false-colored GaAs–GaSb nanowire from the overview image where the GaAs segment is indicated in brown, the GaSb segment in purple, and the catalyst particle in gold. (e) GaSb segment diameter as a function of V/III ratio. The change in V/III ratio was obtained by varying the TMSb flow while keeping the TMGa supply to the growth chamber constant.

In this study we have investigated the interplay between precursor flows, particle composition, and resulting nanowire diameter during Au-seeded GaSb nanowire growth. We have shown that Ga concentration in the particle during axial steady state growth is in the range of 66–94 at. %, while the Sb concentration is in the range of 3–4 at. %. Furthermore, we have demonstrated that the particle volume can enter two distinct regimes depending on the precursor flows used, which affects the resulting nanowire diameter. In the dynamic composition regime, the droplet volume and resulting nanowire diameter were observed to increase dramatically as the TMGa flow was increased or the TMSb flow was decreased, due to increased uptake of Ga atoms in the particle. Using the dynamic response of nanowire diameter to precursor flows, we demonstrated that diameter modulated GaSb nanowires can be obtained. Lastly, we demonstrated a similar correlation between GaSb diameter and V/III ratio in conventional *ex situ* growth. Therefore, this study provides concrete experimental evidence of transferability of the knowledge gained from *in situ* nanowire growths to be applied in conventional *ex situ* growth systems.

## Experimental Methods

### Imaging and Acquisition of in Situ Movies

Nanowire growth was conducted in a Hitachi HF-3300S ETEM instrument equipped with a cold field emission gun operated at 300 kV. The microscope is further equipped with an image aberration corrector (CEOS BCOR). Acquisition of images was performed by using an integrated GATAN OneView IS camera.

### XEDS Acquisition and Analysis

To obtain XEDS spectra, the microscope is equipped with an Oxford Instruments SDD X-Max^N^ 80T system. Spectra were recorded in TEM mode by converging the probe to a 10–20 nm diameter spot and positioning it on the liquid nanoparticle or the as-grown solid nanowire segment. The spectra were recorded with a sampling time of 120 s with 0–20 keV energy range. Quantification of the spectra was carried out using Aztec software (version 3.3) using the Cliff–Lorimer method with the built-in theoretical *k*-factors.

### Sample Holder and Heating Chips

All experiments demonstrated in this article were conducted on a custom-built double tilt holder from Hitachi Inc. Microelectro-mechanical-system (MEMS)-based heating chips supplied by Norcada Inc. were used for *in situ* ETEM growth. The central area of the chip consists of 19 openings surrounded by a thin, electron transparent SiN_*x*_ membrane onto which the Au nanoparticles seeding the growth are deposited.^40^ The central area with 19 openings is surrounded by a tungsten heating coil, which allows uniform Joule heating of the central area for temperatures up to 1100 °C. Temperature was controlled using the Blaze software supplied by Hitachi Inc. using a constant resistance calibration mode.

To determine temperature stability across all used MEMS heating chips, XEDS spectra were acquired with the chips heated to different temperatures around the growth temperature with the beam blanked. From these spectra strobe peak position was determined and compared between the different chips to determine if the temperature was consistent.^41^ The temperature set in the Blaze software controlling the heating was adjusted to match the position of the strobe peaks across all experiments.

### Nanowire Growth and Precursor Supply

The Hitachi HF-3300S ETEM is interfaced with a metal–organic chemical vapor deposition (MOCVD) system where the metalorganics and hydrides used for nanowire growth are supplied via a side port injector on the side of the microscope column. The supply of precursors was controlled using mass-flow controllers (MFCs).

For the growth of GaAs nanowire stems, arsine (AsH_3_) and trimethylgallium (TMGa) were used as precursors with H_2_ being used as a carrier gas for TMGa at a nominal temperature of 420 °C. Details pertaining to the growth of GaAs can be found elsewhere.^42^ In order to form the GaAs–GaSb heterostructures the gas supply to the microscope was stopped and the temperature was reduced to 200 °C. The reason for the reduction in temperature was twofold: the low temperature prevents etching of the nanowire stems and simultaneously prevents further axial growth so that the extent of the ternary transition region can be suppressed.^43^ The group V line of the integrated MOCVD system was purged of the remaining AsH_3_ using N_2_ bypassing the microscope column. Thereafter, TMGa and trimethylantimony (TMSb) with H_2_ as the carrier gas were supplied to the microscope column. A waiting time of 10–15 min was implemented to ensure that a stable pressure and vapor phase composition in the column is achieved. This was done by monitoring pressure transducers (PTs) and signal in the residual gas analyzer (RGA) mounted in the exhaust of the microscope column. After stable gas composition was achieved, the temperature was increased to 420 °C and nanowire growth resumed. Due to residual AsH_3_ in the column, the nanowires underwent a gradual compositional change from GaAs to GaSb via a ternary GaAs_*x*_Sb_1–*x*_ segment. Details on the partial pressures used for the TMGa and TMSb series can be found in Supporting Information SI-1. More details about the *in situ* setup can be found elsewhere.^41,44^

### Conventional MOCVD System

For *ex situ* growth an Aixtron 3 × 2 in. close coupled showerhead (CCS) reactor was used. Size selected Au aerosol particles^40^ with a nominal diameter of 20 nm were deposited onto epiready GaAs ⟨1̅1̅1̅⟩ wafers at an aerial density of 1 μm^–2^ and used as seeds. The process pressure was set to 10 kPa at a total carrier gas flow of 8 slm. The substrates were annealed for 7 min at a set temperature of 630 °C under an hydrogen/arsine (H_2_/AsH_3_) atmosphere with an AsH_3_ partial pressure of *p*_AsH_3__ = 25 Pa before setting the growth temperature of 510 °C. The growth of a GaAs stem, nucleation, and growth of the first GaSb were carried out by supplying the precursors TMGa and TMSb and AsH_3_ with partial pressures in the ranges of *p*_TMGa_ = (1.17–2.35) × 10^–1^ Pa, *p*_TMSb_ = (3.52–6.37) × 10^–1^ Pa, and *p*_AsH_3__ = 5.6 Pa, respectively, with growth times of 3, 0:15–1, and 20 min, respectively. The second GaSb segment where conditions were varied in order to compare to the *in situ* experiments was grown for 20 min with TMSb partial pressures of *p*_TMSb_ = (0.58–3.52) × 10^–1^ Pa. By interrupting the precursor supply, the growth was terminated, and cooling down happened under an H_2_ atmosphere only.

## Supporting Information Available

Details for growth conditions including partial pressures for XEDS and diameter series can be found in SI-1. Quantified XEDS data for the TMGa and TMSb series can be viewed in SI-2, and an example spectrum can be viewed in SI-3. A high-magnification HRTEM image of the GaAs segment where overgrowth was observed at high TMSb flow can be viewed in SI-5. For the diameter modulated nanowires approximate positions along the length of the nanowires where different growth conditions were used are shown in SI-4. Lastly, the growth conditions used in an *ex situ* diameter study with used partial pressures can be viewed in SI-6. (PDF)
