# Supplementary material for: Diameter Control of GaSb Nanowires Revealed by In Situ Environmental Transmission Electron Microscopy
Source: J Phys Chem Lett. 2023 Aug 11;14(33):7404–10. doi: 10.1021/acs.jpclett.3c01928 (PMC10461298; doi:10.1021/acs.jpclett.3c01928)
Supplement: Supplementary file 1 — jz3c01928_si_001.pdf [file jz3c01928_si_001.pdf]

Supporting Information: Diameter Control of GaSb Nanowires Revealed by In-situ Environmental Transmission Electron Microscopy

Mikelis Marnauza[1,2*], Robin Sjökvist[1,2*], Sebastian Lehmann[2,3], Kimberly A. Dick[1,2]

[1]Centre for Analysis and Synthesis, Lund University, 22100 Lund, Sweden

[2]NanoLund, Lund University, 22100 Lund, Sweden

[3]Solid State Physics, Lund University, Box 118, S-221 00 Lund, Sweden

* Both authors contributed equally to this work

SI-1: Growth conditions used in XEDS and diameter series

Table 1. Growth conditions used during the TMGa series.

V/III ratio	TMGa partial pressure, Pa	TMSb partial pressure, Pa	Total reactor pressure, Pa	Notes
11.8	4.93E-03	5.80E-02	3.59E+00	
14.7	4.25E-03	6.24E-02	3.66+00	
19.6	3.41E-03	6.69E-02	3.70E+00	
29.4	2.49E-03	7.31E-02	3.80E+00	
58.7	1.38E-03	8.08E-02	2.69E+00	No axial growth

Table 2. Growth conditions used during the TMSb series.

V/III ratio	TMGa partial pressure, Pa	TMSb partial pressure, Pa	Total reactor pressure, Pa
6.7	3.33E-03	2.24E-02	1.67E+00
13.5	2.94E-03	3.96E-02	2.37E+00
20.2	2.82E-03	5.69E-02	3.13E+00
27.1	2.66E-03	7.22E-02	3.79E+00
33.9	2.58E-03	8.73E-02	4.46E+00
40.6	2.51E-03	1.02E-01	5.12E+00

SI-2: XEDS quantification data for TMGa and TMSb series

Table 3. XEDS quantification data for the TMGa and TMSb series.

Series label	V/III ratio	Ga at.%	Sb at.%	Au at.%	Notes
Sb series	6.7	94.35	3.41	2.24	
	13.5	91.52	4.55	3.93	
	20.2	81.71	4.07	14.22	
	27.1	69.89	3.01	27.10	
	33.9	66.48	3.67	29.84	
	40.6	67.17	3.32	29.51	
Ga series	11.8	93.26	4.69	2.05	
	14.7	93.01	3.92	3.06	
	19.6	87.77	3.58	8.65	
	29.4	69.53	4.22	26.25	
	58.7	53.93	4.94	41.14	No axial growth

SI-3: XEDS spectra of the catalyst particle during growth

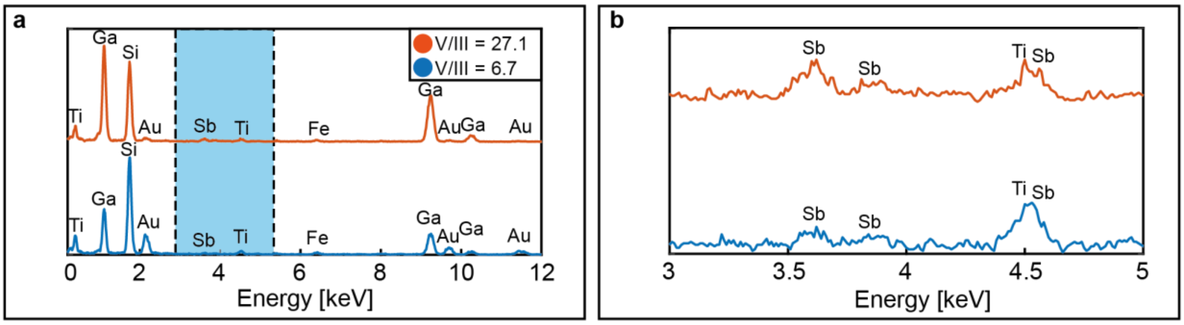

Figure S1. XEDS spectra of the catalyst particle during growth. (a) XEDS spectra with major peaks marked for the catalyst particle at different V/III ratios. The cyan shaded region marks the region shown in panel (b) where the Sb L family peaks used in quantification are shown. Elements other than Ga, Sb and Au arise in the spectra due to the holder, microscope and MEMS chip components.

SI-4: Diameter modulated nanowires with illustrated changes in V/III ratio

For the diameter modulated nanowires from Figure 3 in the main text we here show approximate positions along the length of the nanowire where certain growth conditions were used (see Figure S2). Note that presence of slight radial overgrowth alters the diameter over time and the different segments were not grown for the same amount of time, resulting in segments of different length. The start of a steady state growth regime for each of the used growth conditions was determined by acquiring XEDS spectra and measuring nanowire diameter at the growth interface.

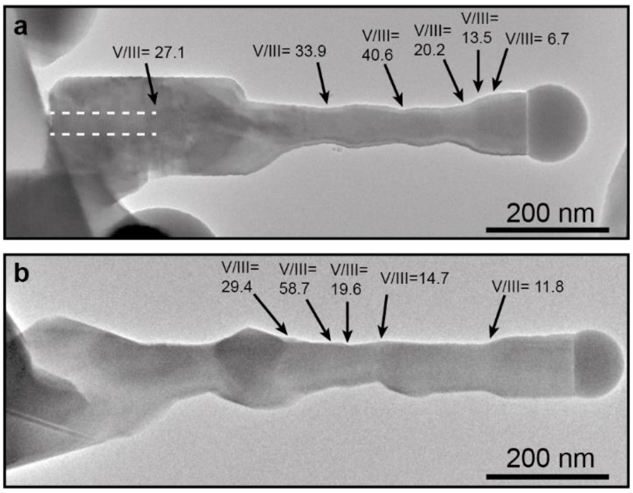

Figure S2. Diameter modulated nanowires imaged after growth. (a) The resulting nanowire after recording the TMSb series. (b) The resulting nanowire after recording the TMGa series. The approximate positions of used growth conditions along the nanowire length are indicated by arrows in each of the respective images.

SI-5: Image of GaAs segment overgrowth at high TMSb flow

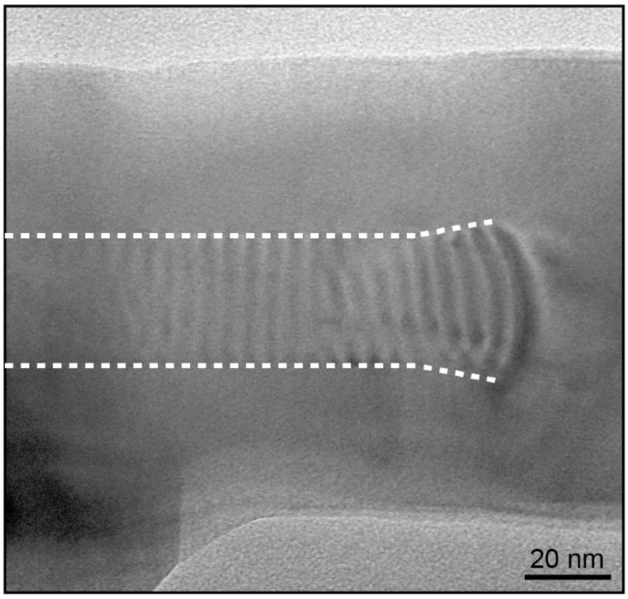

Figure S3. High magnification HRTEM image of the overgrown GaAs nanowire stem from the nanowire depicted in the main text Figure 3 (b). The white dashed outline marks the original GaAs nanowire stem, which can also be seen due to the presence of Moiré fringes caused by the radial overgrowth of GaSb on the GaAs nanowire.

SI-6: Growth conditions used in ex-situ diameter study

Table 4. Growth conditions used during the GaSb segment growth in ex-situ growths.

V/III ratio	TMGa partial pressure, Pa	TMSb partial pressure, Pa
0.5	1.17E-01	5.84E-02
1.0	1.17E-01	1.17E-01
2.0	1.17E-01	2.34E-01
3.0	1.17E-01	3.52E-01
